# Effects of Sublethally Injured Campylobacter jejuni in Mice

**DOI:** 10.1128/spectrum.00690-22

**Published:** 2022-07-11

**Authors:** Gayani Weerasooriya, Andrea R. McWhorter, Samiullah Khan, Kapil K. Chousalkar

**Affiliations:** a School of Animal and Veterinary Sciences, The University of Adelaide, Roseworthy, South Australia, Australia; University of Maryland Eastern Shore

**Keywords:** *Campylobacter jejuni*, chicken, mice, virulence

## Abstract

Globally, Campylobacter spp. are the most common food-associated bacterial cause of human gastrointestinal disease. Campylobacteriosis is primarily associated with the consumption of contaminated chicken meat. Chemical decontamination of chicken carcasses during processing is one of the most effective interventions to mitigate Campylobacter contamination. Following exposure to sanitizers, however, sublethally injured populations of bacteria may persist. The risk that sublethally injured Campylobacter pose for public health is unknown. Furthermore, the virulence potential of sublethally injured Campylobacter jejuni during prolonged storage in relation to host pathogenesis and the host immune response has not been well established. Therefore, we evaluated the effects of sublethally injured C. jejuni on the host, after storage in chicken meat juice. C57BL/6 mice were infected with two C. jejuni chicken meat isolates or the ATCC 33291 strain that had been stored in the chicken meat juice, after exposure to chlorine or acidified sodium chlorite (ASC). Although chlorine exposure was unable to reduce intestinal colonization by C. jejuni, exposure to ASC significantly reduced the intestinal colonization and tissue translocation in mice. The expression of pro- and anti-inflammatory cytokine genes for interleukin-6 (*IL-6*), *IL23a*, and *IL-10*, Toll-like receptor 2 (*TLR2*) and *TLR4* genes, and host stress response genes (*CRP*, *MBL1*, and *NF-κB1*) were significantly reduced following the exposure to ASC. Our results demonstrated that sublethally injured C. jejuni has reduced virulence potential and colonization in mice. The data contribute toward clarification of the importance of chemical decontamination during processing to minimize human campylobacteriosis.

**IMPORTANCE** Campylobacter is the most common cause of bacterial gastrointestinal disease, and consumption of contaminated poultry is frequently identified as the source of bacteria. The survivability and virulence potential of sublethally injured Campylobacter following exposure to chemicals which are commonly used to eliminate Campylobacter during the poultry meat processing are of concern to the food industry, government health officials, and consumers. Here, we demonstrate that sublethally injured Campylobacter jejuni has reduced bacterial virulence and colonization potential in mice.

## INTRODUCTION

Campylobacteriosis is the most common bacterial foodborne human gastrointestinal disease. The genus Campylobacter consists of 27 species and 8 subspecies, but C. jejuni and C. coli are the primary causal agents for foodborne gastroenteritis (1), which can also lead to immunological disorders such as Guillain Barré syndrome, flaccid paralysis, or Miller Fisher syndrome (2, 3). As a foodborne pathogen, Campylobacter can be transmitted via various types of foods as well as water (4). Consumption of undercooked chicken meat, however, is frequently identified during traceback investigation as the primary source of Campylobacter (5). In humans, colonization and clinical disease can manifest following exposure to a small infectious dose (100 to 5,000 organisms) (6). Therefore, reducing Campylobacter load on chicken meat during processing is an important component of food safety.

Campylobacter contamination of chicken meat in processing plants through to point of sale remains significant (7, 8). Generally recognized as safe (GRAS) sanitizers, such as chlorine (sodium hypochlorite), acidified sodium chlorite (ASC), and peracetic acid (PAA), are often used in spin chill tanks as well as in pre- and postdipping tanks to mitigate Campylobacter levels on chicken meat during processing (9–11). Campylobacter is sensitive to environmental stress and is known to be fastidious, but viable bacteria persist in the chicken meat supply chain (12). Thus, the presence of sublethally injured C. jejuni on chicken meat may increase the risk of cross-contamination (13–15). In particular, bacteria in residual chicken meat juice (CMJ) collected during meat packaging could potentially increase the cross-contamination in the kitchen encountered with poor hygienic practices (16, 17). While Campylobacter lacks the global stationary-phase response factor (RpoS) (18), C. jejuni induces different stress resistance mechanisms that enable survivability in the food chain, including the viable but nonculturable state (VBNC) and an adaptive tolerance response (ATR) (19). Furthermore, exposure to various stresses, such as oxidative stress, starvation, cold stress, and acidic stress, induces virulence in C. jejuni (15, 20). Following exposure to sanitizers, sublethally injured C. jejuni expresses both virulence and stress response genes and retains the capacity to invade cultured intestinal cells *in vitro* (21).

Despite the prevalence of C. jejuni as a main foodborne pathogen, knowledge of C. jejuni pathogenesis and host immune response is comparatively limited (22). Numerous *in vitro* experiments have demonstrated that C. jejuni can invade intestinal epithelial cells and can survive inside both phagocytic and epithelial cells (23, 24). Despite the limitations for natural resistance of C. jejuni in colonization (25), systemic infections with extraintestinal tissue invasion have been demonstrated in mouse models (26, 27). A previous *in vivo* study demonstrated the invasion potential of sublethally injured C. jejuni and the expression of virulence genes during prolonged storage in chicken meat juice (21). However, the virulence potential and pathogenicity of sublethally injured C. jejuni following exposure to sanitizers during processing is not well understood. In this study, we investigated the virulence potential of C. jejuni, stored in chicken meat juice at refrigeration temperature following exposure to either chlorine or ASC, in a C57BL/6 mouse model. In addition, we demonstrated an induced immune response and host stress response in mice during Campylobacter infection in a gene expression study.

## RESULTS

### Culturability of Campylobacter jejuni from chicken meat juice.

Suspensions of each Campylobacter isolate (ATCC 33291, C5, and C10) were prepared for each treatment group. Bacteria were subsequently treated with chlorine or ASC before inoculation into chicken juice. C. jejuni counts in the CMJ were determined on day 0 and day 5 to verify the inoculum. The bacterial count on day 0 in untreated groups of ATCC 33291, C5, and C10 was 2 × 10^10^, 4 × 10^10^, and 1 × 10^10^ CFU/mL, respectively, while chlorine-treated ATCC 33291, C5, and C10 groups consisted of 2 × 10^8^, 2 × 10^9^, and 8 × 10^5^ CFU/mL. The Campylobacter counts of the inoculum on day 5 of the untreated groups ATCC 33291, C5, and C10 were 2 × 10^9^, 4 × 10^9^, and 1 × 10^9^ CFU/mL, respectively. The chlorine-treated ATCC 33291, C5, and C10 groups had 7 × 10^6^, 2 × 10^7^, and 5 × 10^4^ bacterial loads, respectively. For all the ASC-treated groups, C. jejuni was not culturable at either day 0 or day 5. A previous study showed that C. jejuni isolates respond differently to the same concentration of sanitizers (28) and that the survivability of sublethally injured C. jejuni in chicken meat is strain dependent (21). Therefore, although the different treatment groups had different bacterial counts, mice were administered a dose based on the original inoculum before sanitizer exposure to mimic a kitchen cross-contamination event. This enabled characterization of the virulence potential of sublethally injured C. jejuni in CMJ after 5 days of storage.

### Clinical infection in C57BL/6 mice.

**FIG 1:**
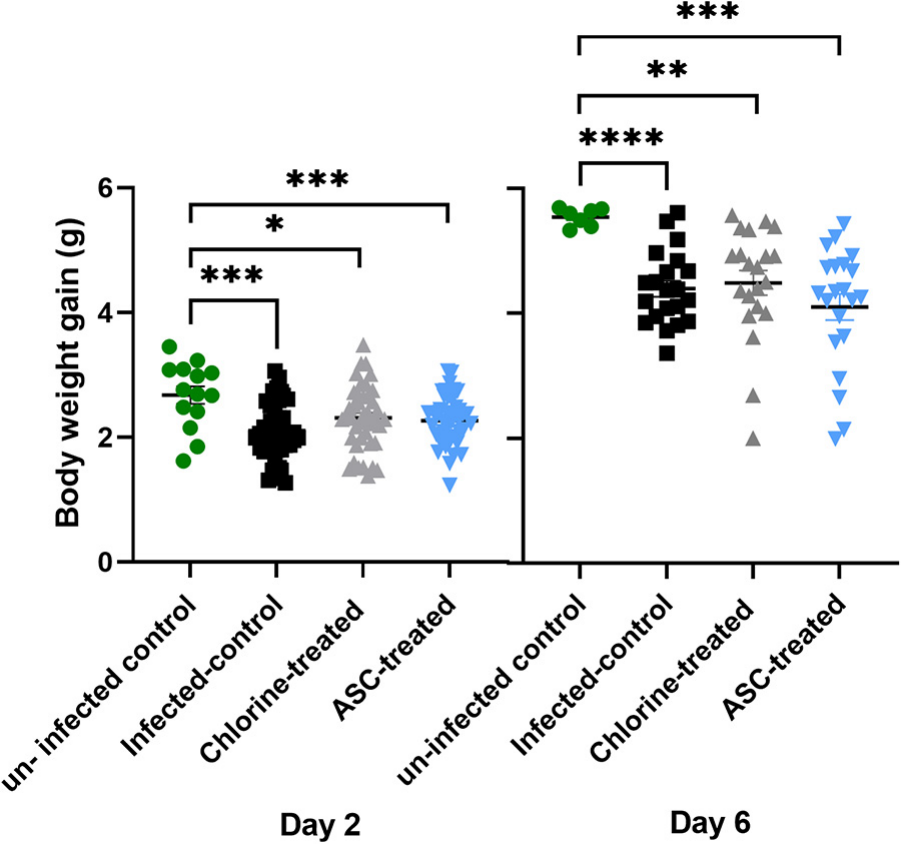
Body weight gain (g) of mice on day 2 p.i. and day 6 p.i. The body weight gains of infected-control (black), chlorine-treated (gray), and ASC-treated (blue) groups were compared with that in the uninfected-control group (green). The body weight gains of individual mice were calculated by subtracting the body weight either on day 2 p.i. or day 6 p.i. from the day 0 body weight. The body weight gain of all infected groups was significantly lower than in the noninfected control group. *, *P* ≤ 0.01; **, *P* ≤ 0.001; ***, *P* ≤ 0.0001.

No clinical signs of disease were observed postinfection. Additionally, a postmortem examination revealed no gross evidence of clinical infection lesions or inflammation in any of the C. jejuni-infected mice. On day 2 and day 6 postinfection (p.i.), mice infected with C. jejuni exhibited a significantly (*P* ≤ 0.01) lower body weight gain compared with the uninfected control group (Fig. 1). No significant difference in body weight was observed between the C. jejuni treatment groups at other time points.

### Fecal hemoglobin ELISA.

**FIG 2:**
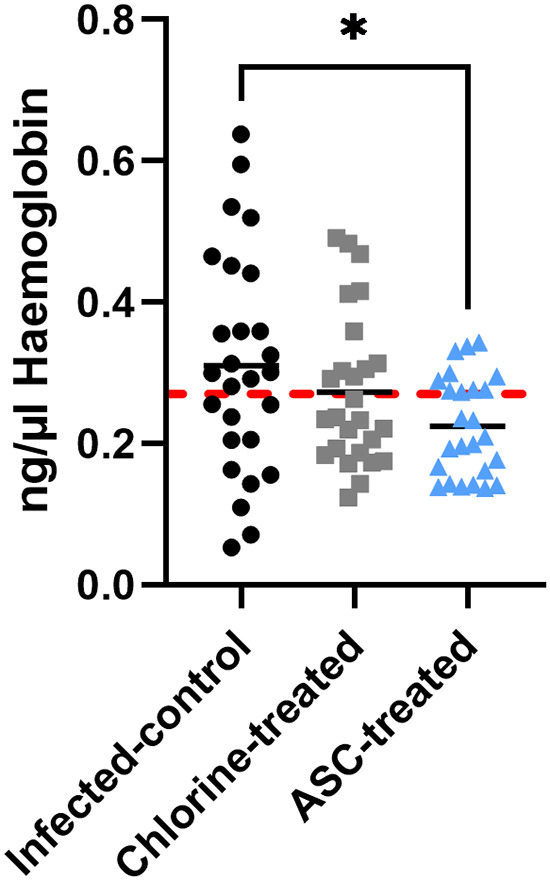
Fecal hemoglobin levels in infected mice. The hemoglobin was measured in the fecal pellets, collected as pooled samples from a cage relevant to the group, on day 3 and day 7 p.i. As no statistically significant effect of time was detected, the hemoglobin data for fecal samples collected on day 3 and day 7 were combined to test for the effect of the treatment. The hashed line (red) indicates the limited detection of the uninfected control group. *, *P* ≤ 0.01.

Fecal hemoglobin was measured in an enzyme-linked immunosorbent assay (ELISA) to characterize C. jejuni-induced enteritis (Fig. 2). No statistically significant effect of infection time was detected. Therefore, the data for fecal samples collected on both day 3 and day 7 were pooled to test for the effects of the treatment. The mean concentration of hemoglobin for the infected-control group was 0.31 ± 0.02 ng/μL (mean ± standard error of the mean [SE]), which was significantly higher (*P <* 0.05) than in the ASC-treated group (0.22 ± 0.01 ng/μL) (Fig. 2). Although the hemoglobin level was lower in the chlorine-treated group (0.27 ± 0.02 ng/μL) than in the infected-control treated group, it was not statistically significant.

### Fecal shedding and tissue colonization of C. jejuni in infected mice.

Using direct bacterial culture methods, all mice were Campylobacter negative prior to commencing the infection challenge. On days 3 and 7 p.i., all fecal and tissue samples were culture negative upon direct plating on modified charcoal-cefoperazone-deoxycholate (mCCDA) agar plates. Following bacterial enrichment, no culturable C. jejuni was detected in any of the fecal or tissue samples collected.

### C. jejuni species-specific PCR in fecal and tissue DNA.

C. jejuni species-specific PCR was performed using fecal and tissue DNA to detect colonization by possibly sublethally injured Campylobacter. Fecal samples were collected from cages as a pooled sample, relevant to the groups (ATCC 33291, C5, and C10) to determine fecal shedding and colonization on day 3 p.i. (*n *= 20) and day 7 p.i. (*n *= 10).

Of six pooled fecal samples from six separate infected-control groups (cages), five were PCR positive (83.33%) on day 3, while a pooled fecal sample of group C10 was negative. Fecal samples from six chlorine-treated groups (cages) were PCR positive (100%) on day 3, while all samples from the six ASC-treated groups were PCR negative (0%) on day 3. On day 7, all fecal samples from the remaining three infected-control, three chlorine-treated, and three ASC-treated groups (cages) were PCR positive (100%). All the pooled fecal samples of uninfected control groups on both days 3 and 7 p.i. were PCR negative.

**FIG 3:**
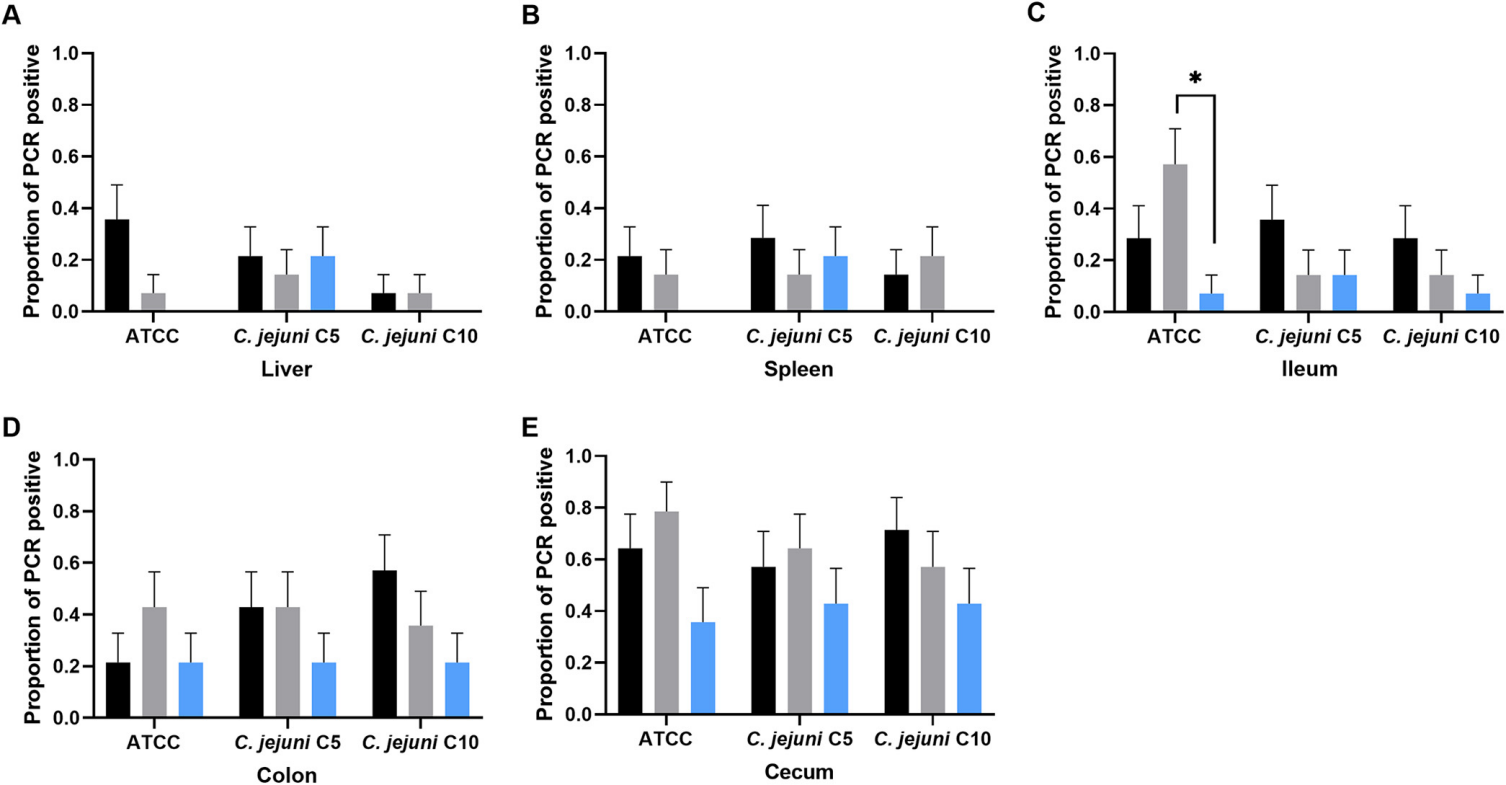
Proportion of C. jejuni positive tissue samples by species-specific PCR. The PCR-positive liver (A), spleen (B), ileum (C), colon (D), and cecum (E) tissues are presented as a proportion of the total number of respective tissue samples in the infected-control (black), chlorine-treated (gray), or ASC-treated (blue) group. The proportion of positive samples was not significantly different between day 3 and day 7 p.i.; therefore, the data were pooled to assess the effect of treatment. The colonization results data are means ± SE for each tissue type. *, *P* ≤ 0.01.

All tissue samples collected on day 3 and day 7 p.i. were screened for C. jejuni colonization using the Campylobacter species-specific PCR (Fig. 3). No significant difference in the proportion of positive samples was observed between days 3 and 7 p.i.; therefore, the data were pooled to assess the effect of treatment in the infected-control, chlorine-treated, and ASC-treated groups. Although a PCR method that permits the differentiation between live and dead cells was not used, not all infected mice were PCR positive. Therefore, we assumed that the confirmed PCR-positive samples represented tissue colonization. Regardless of the type of tissue or the C. jejuni strain, lower colonization was observed in ASC-treated groups than in chlorine-treated or infected-control groups. This was most evident in the ileum, where the proportion of PCR-positive samples in the ASC-treated ATCC 33291 group was significantly lower (*P* ≤ 0.05) than that in the chlorine-treated group. In the liver and spleen, except for C. jejuni C5, all other ASC-treated groups were PCR negative.

C. jejuni colonization was not significantly different between chlorine-treated and infected-control groups in either colon or cecum. A higher proportion of C. jejuni PCR-positive samples was recorded for ileum, cecum, and colon samples compared to spleen and liver samples. In the chlorine-treated groups, the proportion of positive colon and cecum samples (Fig. 3D and E) for ATCC 33291 (0.60 ± 0.09), C5 (0.53 ± 0.09), and C10 (0.46 ± 0.09) groups, respectively, were significantly higher (*P* ≤ 0.01) than in the spleen and liver (Fig. 3A and B). Increased colonization with higher positive proportions was observed in the ileum of the chlorine-treated ATCC 33291 group (0.57 ± 0.13) compared to the C5 (0.14 ± 0.09) and C10 groups (0.14 ± 0.09).

### Expression of inflammatory cytokines.

**FIG 4:**
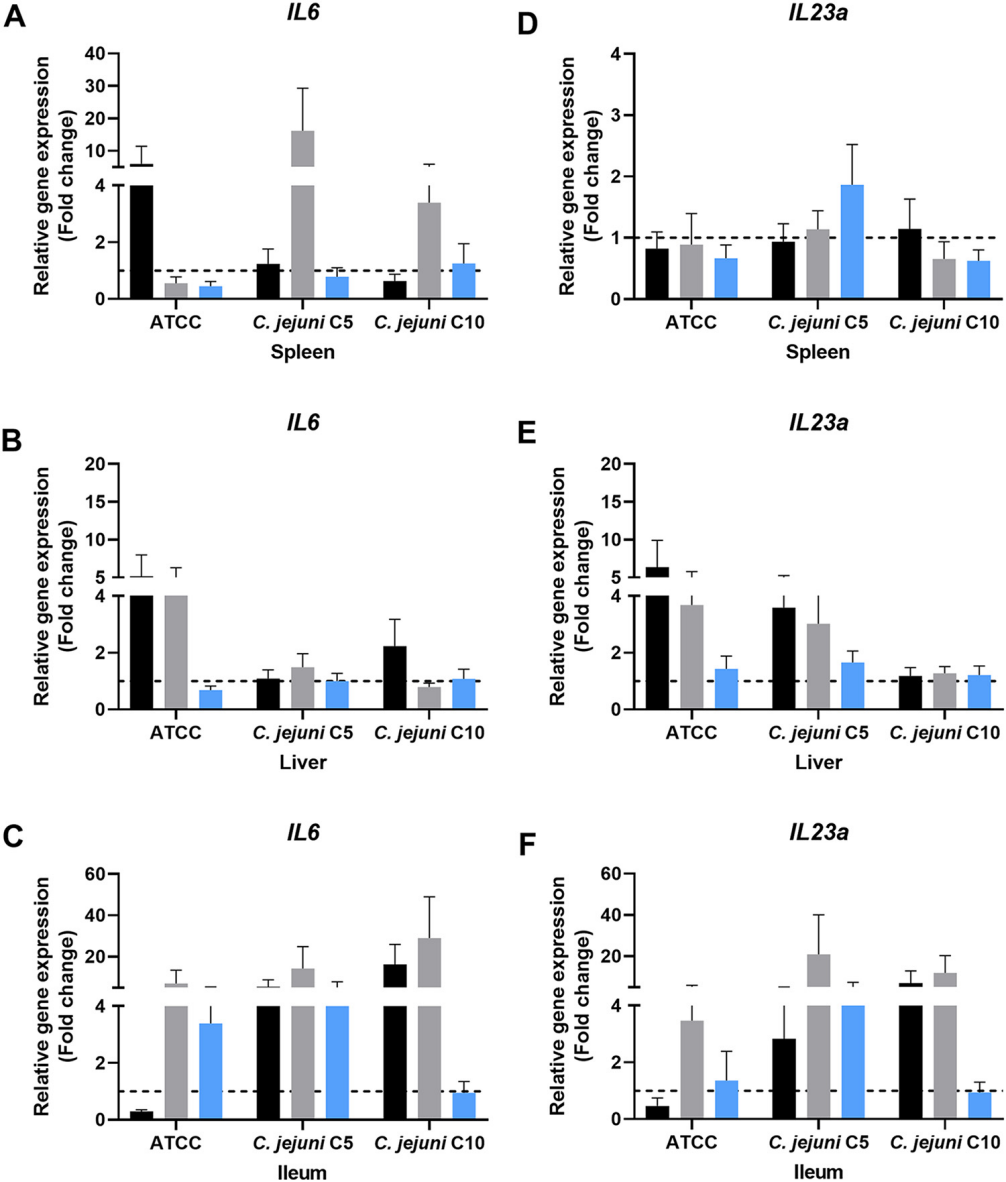
Expression of proinflammatory cytokine genes *IL-6* and *IL-23a* in mice. (A to C) *IL-6* expression in spleen (A), liver (B), and ileum (C). (D to F) *IL-23a* expression in spleen (D), liver (E), and ileum (F). Bars in each graph show infected-control (black), chlorine-treated (gray), and ASC-treated (blue) groups. Gene regulation of chlorine- or ASC-exposed C. jejuni infection in mice was assessed using the untreated control group as a reference. The hashed line indicates the baseline for gene regulation and any value below, it shows downregulation, while any value above, it shows upregulation.

**FIG 5:**
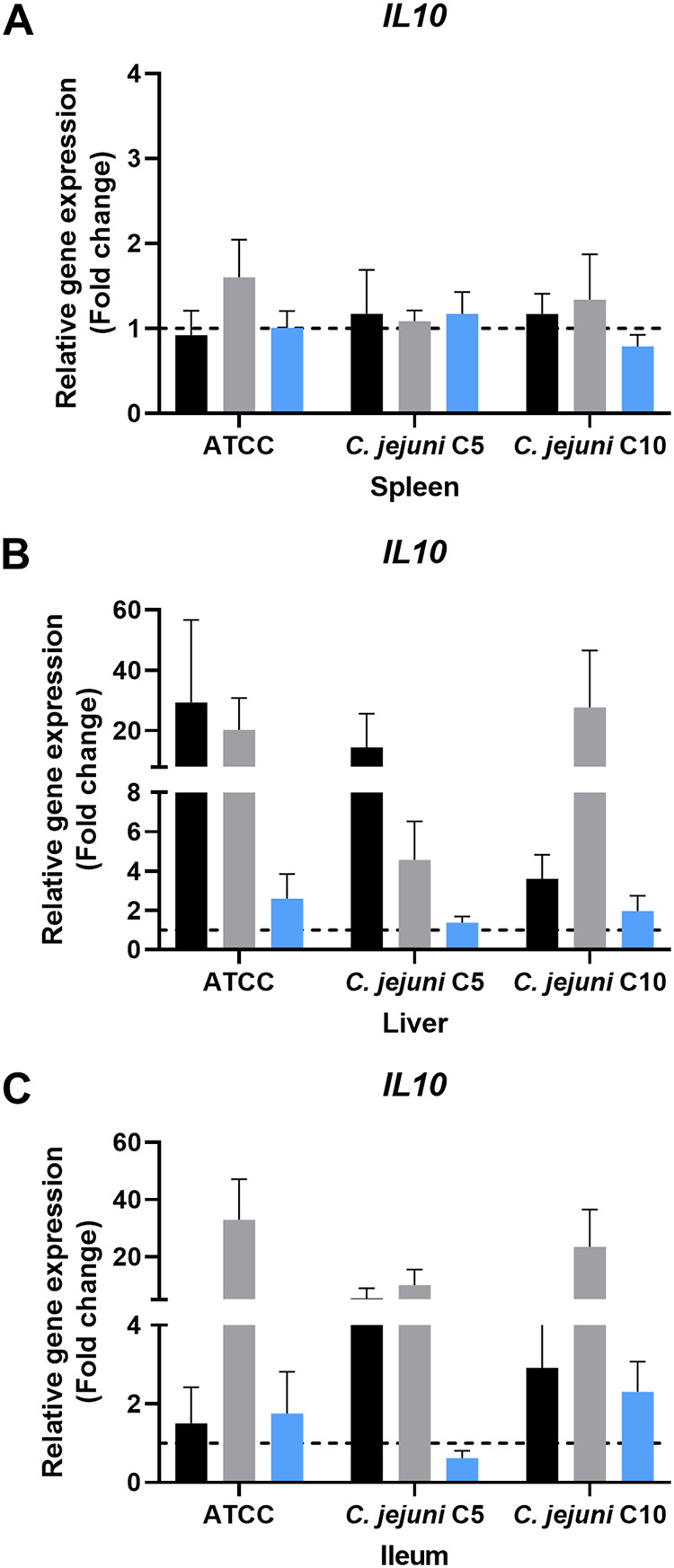
Expression of the anti-inflammatory cytokine gene *IL-10* in mice. *IL-10* expression in spleen (A), liver (B), and ileum (C) in infected-control (black), chlorine-treated (gray), and ASC-treated (blue) groups. Gene regulation of chlorine- or ASC-exposed C. jejuni infection in mice was assessed using the untreated control group as a reference. In individual graphs, the hashed line indicates the baseline for gene regulation and any value below, it shows downregulation, while any value above, it shows upregulation.

The stimulation of host immune response genes was also investigated. In the present study, the expression of the proinflammatory cytokine genes for interleukin-6 (*IL-6*) (Fig. 4A to C) and *IL-23a* (Fig. 4D to F) and anti-inflammatory cytokine gene *IL-10* (Fig. 5A to C) was studied. Activation of the innate immune system during C. jejuni infection induced proinflammatory and anti-inflammatory cytokines.

*IL-6* was significantly (*P* ≤ 0.01) upregulated in the spleen and liver of the ATCC 33291 infected-control group compared to the C5 and C10 groups (Fig. 4A and B). In the spleen, *IL-6* was significantly (*P* ≤ 0.01) upregulated in the chlorine-treated C5 group compared to the other treatment groups (Fig. 4A). *IL-6* was downregulated in the liver in all other chlorine-treated groups except for the ATCC 33291-infected group (Fig. 4A and B). The *IL-6* expression levels were significantly higher (*P* ≤ 0.05) in the ileum than in the spleen and liver in all other infected-control, chlorine-treated, and ASC-treated groups. However, it was downregulated in the infected-control ATCC 33291 and ASC-treated C10 (Fig. 4C) groups. *IL-6* was upregulated in the infected-control C5 and C10 groups, with fold changes of 5.07 ± 2.1 and 4.33 ± 1.9, respectively (Fig. 4C). Interestingly, the chlorine-treated group had the highest expression levels, with an average fold change of 22.01 ± 6.10 compared to the infected-control and ASC-treated groups in the ileum.

*IL-23a* was downregulated in the spleen of all the treatment groups except for the ASC-treated C5 group (Fig. 4D). The expression of *IL-23a* in the infected-control and chlorine-treated ATCC 33291 and C5 groups was high in the liver, while it was downregulated in all the ASC-treated groups (Fig. 4E). Similar to *IL-6*, the expression level of *IL-23a* in the ileum was higher than in other tissue types in the infected-control and chlorine-treated groups, except in the infected-control ATCC 33291 group (Fig. 4F). Notably, the expression of *IL-23a* was downregulated in all other ASC-treated groups.

The expression level of anti-inflammatory cytokine *IL-10* was not significantly regulated in the spleen (Fig. 5A). *IL-10* was upregulated in the liver in all the infected-control and chlorine-treated groups (Fig. 5B). In assessment of the sanitizer effects, the chlorine-treated group compared to ASC-treated group showed higher expression levels in the liver. The expression of *IL-10* was downregulated in mice challenged with C. jejuni treated with ASC in either the spleen, liver, or ileum, except for a slight upregulation in C10 in the ileum (Fig. 5A to C).

### Expression of Toll-like receptor genes.

**FIG 6:**
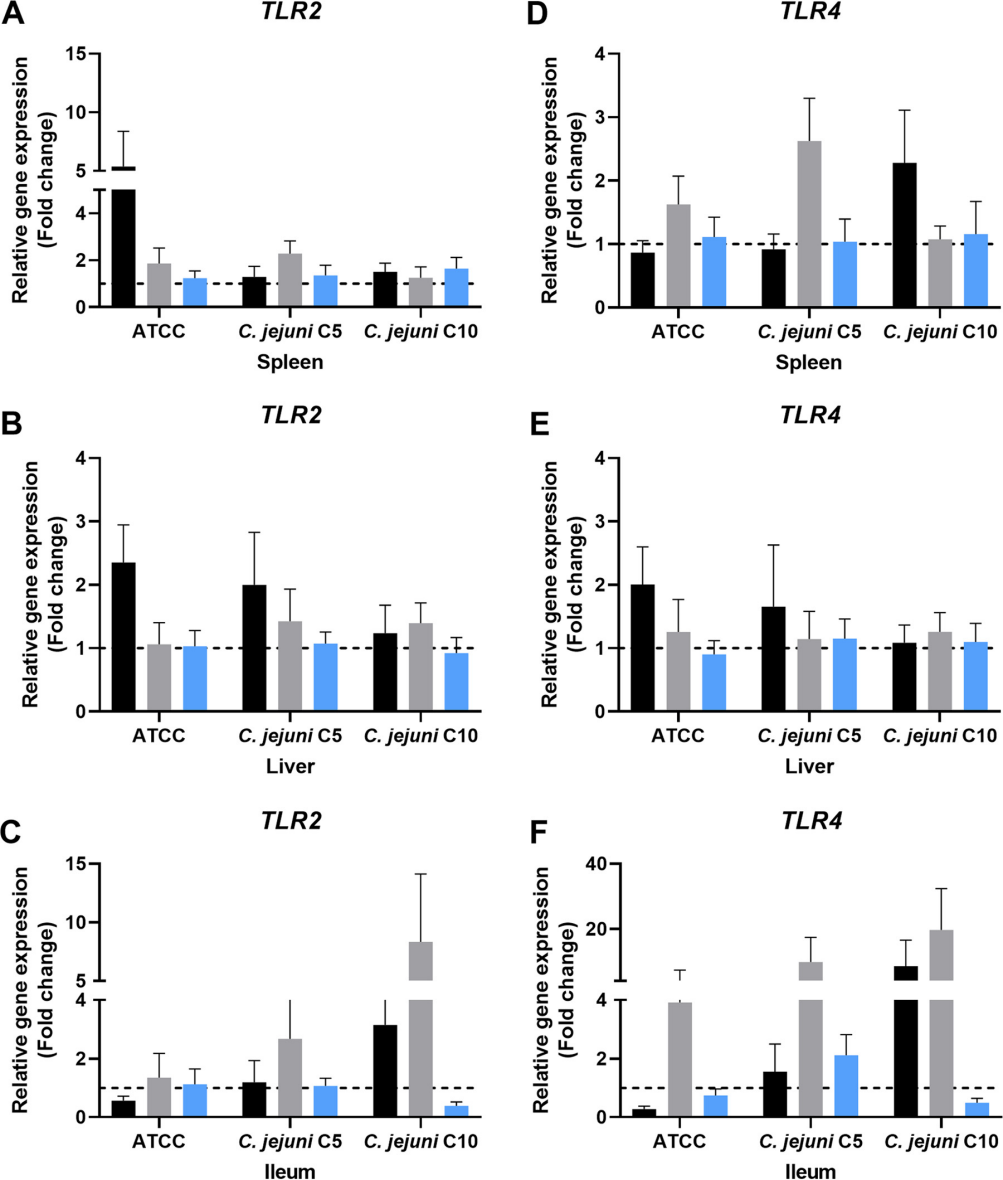
Expression of Toll-like receptor genes *TLR2* and *TLR4* in mice. (A to C) *TLR2* expression levels in spleen (A), liver (B), and ileum (C). (D to F) *TLR4* expression levels in spleen (D), liver (E), and ileum (F). Treatment groups are infected-control (black), chlorine-treated (gray), and ASC-treated (blue) groups. Gene regulation of chlorine- or ASC-exposed C. jejuni infection in mice was assessed using the untreated control group as a reference. In individual graphs, the hashed line indicates the baseline for gene regulation and any value below it shows downregulation, while any value above it shows upregulation.

The expression of *TLR2* (Fig. 6A to C) and *TLR4* (Fig. 6D to F) was studied in the spleen, liver, and ileum of mice infected with sanitizer-exposed C. jejuni in chicken meat juice. The *TLR2* expression in the spleen or liver was not upregulated in any of the infected-control or chlorine-treated groups, except for the spleen in the infected-control C. jejuni ATCC 33291 group (Fig. 6A and B). In the ileum, *TLR2* was upregulated in the chlorine-treated C5 and C10 groups (Fig. 6C). Interestingly, *TLR2* was downregulated in the spleen, liver, and ileum in all ASC-treated groups compared with the chlorine-treated groups (Fig. 6A to C). Unlike *TLR2*, *TLR4* was downregulated in all the tissues of the ASC-treated groups, except in the ileum of C. jejuni C5 (Fig. 6). The expression levels of *TLR4* in the ileum (Fig. 6F) were significantly higher (*P* ≤ 0.05) in the chlorine-treated group compared to the spleen and liver of the same treatment groups (Fig. 6D and E). Furthermore, the observed average expression of *TLR4* in the ileum in the chlorine-treated group was higher (fold change of 11.14 ± 1.23) than in the infected-control group (fold change of 3.47 ± 1.20).

### Expression of host stress response genes.

**FIG 7:**
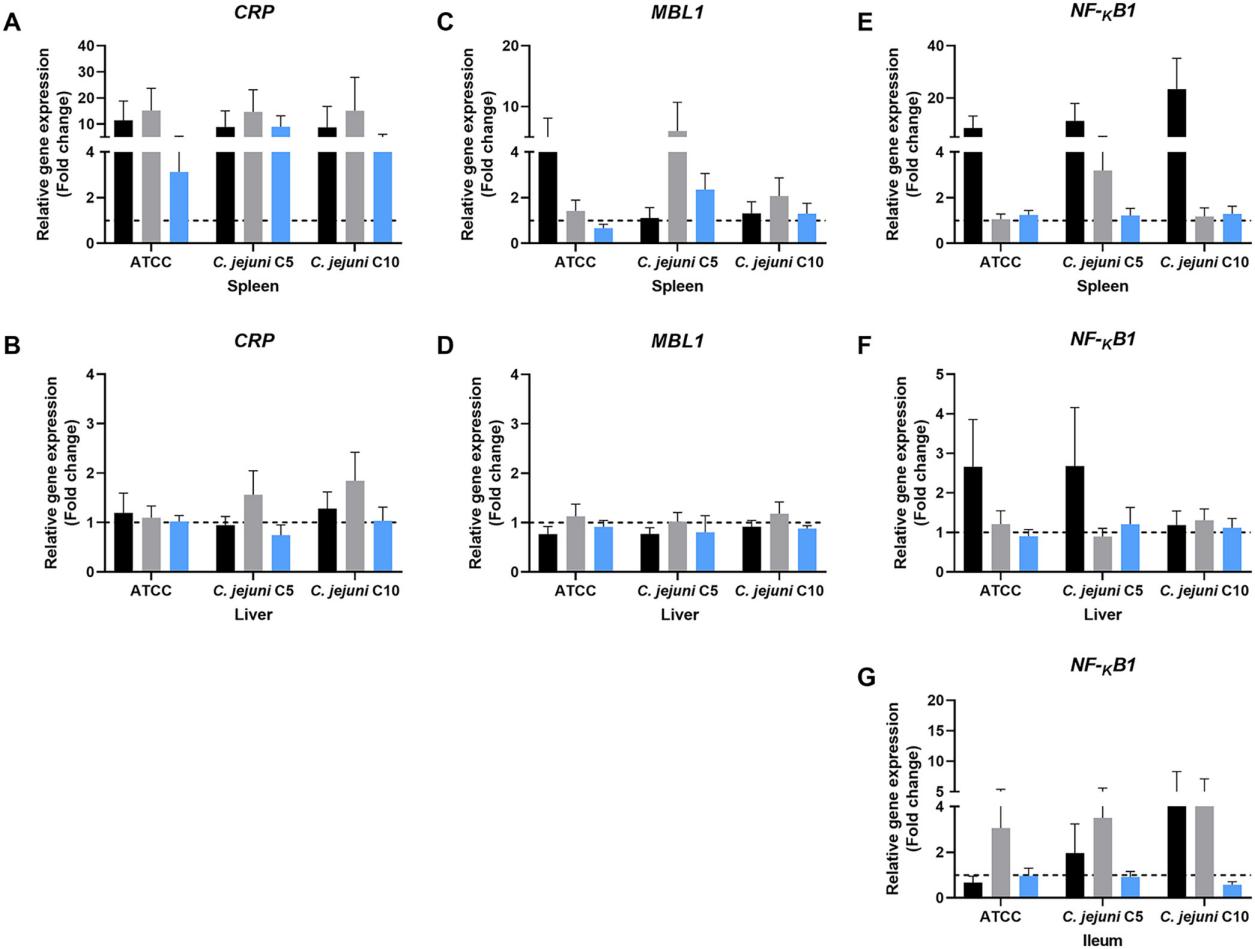
Expression of host stress response genes *CRP*, *MBL1*, and *NF-κB1* in mice. (A and B) *CRP* expression levels in spleen (A) and liver (B). (C and D) *MBL* expression levels in spleen (C) and liver (D). (E to G) *NF-κB1* expression levels in spleen (E), liver (F), and ileum (G). Treatment groups are infected-control (black), chlorine-treated (gray), and ASC-treated (blue). Gene regulation of chlorine- or ASC-exposed C. jejuni infection in mice was assessed using the untreated control group as a reference. In individual graphs, the hashed line indicates the baseline for gene regulation and any value below, it shows downregulation, while any value above, it shows upregulation.

The host stress response is a first-line defense mechanism against infection that acts as an indicator of systemic infection and liver damage. Therefore, we investigated differences in the expression levels of *CRP* (Fig. 7A and B), *MBL1* (Fig. 7C and D), and *NF-κB1* (Fig. 7E to G) to determine if C. jejuni exposed to sanitizers still induced a host stress response in mice.

*CRP* was upregulated in the spleen of all the infected groups, while it was downregulated in the liver of all the groups (Fig. 7A and B). However, in the chlorine-treated C5 and C10 groups, *CRP* was slightly upregulated in the liver. The expression of *CRP* was significantly higher (*P* ≤ 0.05) in the spleen compared to the liver. Although the expression level was higher in the chlorine-treated group, there was no significant difference between the expression levels in the spleen of either in infected-control or ASC-treated groups (Fig. 7A). *MBL1* was slightly upregulated in the spleen in the infected-control C. jejuni ATCC 33291 group and chlorine-treated C. jejuni C5 group (Fig. 7C). *MBL1* was downregulated in the liver of all the infected-control or chlorine-treated groups, while it was downregulated in both spleen and liver of ASC-treated groups (Fig. 7C and D).

The average expression of *NF-κB1* in the spleen was significantly higher (*P* ≤ 0.05) in the infected-control group compared to the chlorine- or ASC-treated groups (Fig. 7E)*. NF-κB1* was upregulated in the liver in infected-control ATCC 33291 and C5 groups, while it was downregulated in all other groups (Fig. 7F)*. NF-κB1* was upregulated in the ileum of the chlorine-treated ATCC 33291 and C5 groups compared to the counterparts in the infected-control groups (Fig. 7G). *NF-κB1* was downregulated in the liver, spleen, and ileum in all the groups challenged with ASC-treated C. jejuni (Fig. 7E to G).

### Expression of C. jejuni virulence genes.

The expression of virulence and stress response genes in sublethally injured C. jejuni is upregulated during prolonged storage in chicken meat juice (21). Therefore, quantitative PCR (qPCR) experiments were conducted to determine the expression levels of virulence genes (*flaA*, *cadF*, *peb1*, *racR*, and *cheA*) in C. jejuni in mouse tissues. However, no measurable expression levels of individual genes were confirmed by qPCR amplification curve, melt curve analysis, or agarose gel electrophoresis. This was likely due to a low bacterial load which was not able to produce RNA in sufficient quantity for assessing gene expression (29).

## DISCUSSION

Campylobacter spp. are the most common foodborne pathogens that cause bacterial enteritis in humans; nevertheless, they are considered the most fastidious and sensitive to environmental stress (30). To mitigate Campylobacter contamination, poultry processing plants often utilize sanitizers on chicken carcasses. Chlorine is one of the most used sanitizers in poultry processing. The comparative bactericidal effects of chlorine on C. jejuni with other sanitizers such as ASC have not been completely explored. One aspect of concern is the persistence of sublethally injured Campylobacter jejuni in chicken meat juice of a sanitized chicken, which is retained in packaging materials and can potentially cross-contaminate other food and/or equipment in the kitchen environment. Sublethally injured bacteria are a significant public health issue (31), but their virulence potential is not completely understood. In this study, we compared the *in vivo* virulence and host response to sublethally injured C. jejuni following exposure to chlorine and ASC, storage in chicken meat juice, and then feeding to C57BL/6 mice.

Clinical symptoms of the disease were not observed for any of the C. jejuni-infected treatment groups. This finding is consistent with other Campylobacter mouse studies (25, 32). An interesting observation was that mice infected with untreated C. jejuni exhibited slower weight gain than either the chlorine- or ASC-treated C. jejuni groups. Untreated C. jejuni-infected mice also exhibited significantly higher fecal hemoglobin, which was likely due to a higher colonization potential than that of bacteria treated with either chlorine or ASC. C. jejuni infection caused blood-tinged stools and weight loss without producing either severe clinical signs or mortality (33). Following exposure to chlorine or ASC, C. jejuni may exhibit a reduced colonization capacity due to the presence of fewer total culturable bacteria (29).

Although C. jejuni was not cultured from fecal samples, C. jejuni was detected using a species-specific PCR on samples from infected groups. The lack of culturable C. jejuni may be linked with the original inoculum. Even though the mice were inoculated with a high bacterial load (10^9^ CFU/mL C. jejuni in infected-control groups), reduced culturability was observed for bacteria treated with chlorine or ASC. The reduced colonization could be a consequence of physiological damage and morphological changes of C. jejuni during prolonged storage in CMJ at refrigerated temperature. Colonization of C. jejuni with low inoculum counts (10^4^ to 10^5^ CFU/mL) was previously reported in a study conducted with viable but nonculturable C. jejuni (34). Furthermore, reduced colonization could be due to the host defense mechanisms such as acidic pH, gastric enzymes, gut microflora, bile and mucus secretions, antimicrobial peptides, or reduced culturable bacterial load (35).

Fecal samples collected from the infected-control and chlorine-treated groups were PCR positive for C. jejuni at day 3 and day 7 p.i., suggesting successful bacterial colonization of mice. It is interesting to note that C. jejuni treated with ASC was not detected in mouse feces on day 3 p.i. Feces from the ASC-treated C. jejuni group, however, were positive on day 7 p.i. This could be due to delayed colonization with ASC-treated C. jejuni, which may be due to structural changes in the bacterial cell membrane. It has been demonstrated that following exposure to ASC, C. jejuni exhibits reduced flagellar activity, metabolic activity, and changes in membrane proteins (29). Additionally, viable but nonculturable bacteria have been shown to have delayed colonization and excretion (36). In the present study, we demonstrated that post-ASC exposure, C. jejuni was not culturable and exhibited delayed shedding in feces.

Tissue colonization of sublethally injured C. jejuni in mice was also investigated in spleen, liver, ileum, colon, and cecum using PCR. Regardless of the type of tissue and the C. jejuni strain, reduced tissue colonization was observed in ASC-treated groups compared to chlorine-treated or infected-control groups. The significantly lower intestinal colonization and tissue translocation of C. jejuni in the ASC-treated groups compared to the infected-control and chlorine-treated groups indicate a lower reviving capability of the bacteria in mice. Previously, we showed that ASC causes oxidative cellular injury in C. jejuni (29), which might have been the cause of reduced intestinal colonization and extraintestinal tissue translocation in the current study.

The higher colonization rate in cecum in the infected-control and chlorine-treated groups showed that C. jejuni was able to revive quickly in the gut of the mice. This could be due to chlorine inducing less damage to the bacteria (28). Our findings are supported by a study that demonstrated that oxidative stress neither affected C. jejuni infectivity nor reduced the load in the mouse liver, in comparison to untreated C. jejuni (37). Notably, increased intestinal colonization of the chlorine-treated ATCC 33291 group of mice could be due to an induced host cell invasion potential of C. jejuni following exposure to chlorine. Increased expression of flagellar genes was reported in C. jejuni under oxidative stress following exposure to H_2_O_2_ and chlorine (29, 38). In the current study, the lower intestinal colonization observed for chlorine-treated C10 (low invasive) could be due to genetic differences in the capacity of this strain to invade intestinal cells.

C. jejuni detected by PCR in the spleen and liver demonstrated its extraintestinal tissue translocation. In general, tissue translocation of C. jejuni in the spleen and liver was not significantly different between the infected-control and chlorine-treated groups, except for chlorine-treated ATCC 33291 and C5 (high invasive), where the colonization was significantly higher. The individual strain variation data for colonization might explain the different degrees of pathogenicity caused by different isolates in the same Campylobacter sp. population.

The ability of sublethally injured C. jejuni to stimulate the host immune response was also investigated. Virulence factors of C. jejuni induce the production of inflammatory cytokines by stimulating the intestinal epithelial cells (39). The significantly higher expression of *IL-6* in the ileum compared to spleen and liver in most of the treatment groups could be due to the observed higher intestinal colonization than tissue translocation. The reduced expression levels of *IL-6* in the ASC-treated compared to infected-control and chlorine-treated groups could be due to either reduced colonization or reduced stimulation due to the structural changes in membrane lipopolysaccharide (LPS) and lipooligosaccharide (LOS) following exposure to ASC. The physiological and structural changes in the bacterial cell wall after exposure to ASC have been previously demonstrated in a transcriptomic analysis (29). The downregulation of *IL-6* in the ileum of ASC-treated C10 (low invasive) could be due to strain variation in invasiveness and expression virulence factors. Increased production of IL-6 in C. jejuni infection in mice has been demonstrated in the colonization of C. jejuni-stressed and normal cells (37, 40). *IL-23a* was also highly expressed in infected groups in the ileum compared to spleen and liver, which could be due to the higher intestinal colonization. IL-23 is a cytokine of antigen-presenting cells that contributes to inducing an adaptive inflammatory response to C. jejuni infection via monocytes and dendritic cells in the intestinal inflammatory site. (41). Upregulation of *IL-23a* only in ASC-treated C5 (high invasive) group spleens could be due to the increased tissue colonization. The involvement of IL-23 in the bacterial clearance mechanism has been shown in C. jejuni-infected IL-23^−/−^ mice (41).

*IL-10* was highly upregulated both in the liver and ileum of the infected-control and chlorine-treated groups, but not in the spleen. The antagonistic effect of IL-10 as an anti-inflammatory factor in C. jejuni infection, as it reduces the expression of proinflammatory cytokine and the resolution of the inflammation, has been described previously (42). The observed reduced expression levels of *IL-6* and *IL-23a* in similar groups could be due to the increased expression of *IL-10* in the liver. A significant increase in IL-10 production in the liver has been demonstrated in Campylobacter-infected BALB/c mice, while IL-6 production was not significant (43). In general, *IL-10* was downregulated or not expressed in ASC-treated groups. This could be due to the reduced colonization and structural cellular damage in C. jejuni following exposure to ASC. Studies have shown that only live bacteria can induce IL-10 production during C. jejuni infection (37). Notably, we observed upregulation of *IL-6* and *IL-23a* in the ileum of the ASC-treated C5 (high invasive) group, whereas *IL-10* was downregulated. Studies have shown that IL-10-deficient mice have increased C. jejuni colonization and enteritis and IL-10 suppresses the clinical infection and production of proinflammatory cytokines (44). Nevertheless, the observed differences in the expression of *IL-6* and *IL-23a* in different organs were not consistent, possibly due to the low versus high invasiveness nature of the isolates and sanitizer effects. These effects were evident in the higher expression levels of *IL-6* and *IL-23a* in the ASC-treated C5 (high invasive) group compared levels in the ATCC 33291 and C10 (low invasive) groups.

To further understand cytokine gene expression in mice that received sublethally injured C. jejuni, the expression of *TLR2* and *TLR4* was studied. Toll-like receptors (TLRs) stimulate the host immune system and induce the cytokine response by recognizing bacterial structures, especially LPS, LOS, lipoprotein, and flagellin (45). Interestingly *TLR2* and *TLR4* expression was correlated with the expression of other cytokines (*IL-6*, *IL-10*, and *IL-23a*). While the expression of TLRs was lower in both the spleen and liver in all the infected-control and chlorine-treated groups, it was significantly higher in chlorine-treated groups than in the control. This could be due to increased flagella and flagellin activity, induced by the adaptive stress response mechanism in C. jejuni following exposure to chlorine (28, 29). Increased expression of TLRs was demonstrated in mouse intestinal colonization with a higher C. jejuni load (46). The expression of *TLR2* and *TLR4* was downregulated in ASC-treated groups in spleen, liver, and ileum, except for the liver in the ASC-treated C5 (high invasive) group, which could be due to reduced colonization or lower stimulation of TLR signaling pathways in mice that received sublethally injured C. jejuni. Compared to live bacteria, killed bacteria result in reduced stimulation of TLR2 and TLR4 *in vitro* (47).

To understand the virulence potential of sublethally injured C. jejuni causing clinical infection, we investigated the expression of the host stress response and defense genes *CRP*, *MBL1*, and *NF-κB1*. CRP is expressed in the initial acute-phase systemic response of the host to infection, inflammation, or tissue injury (48), while MBL plays a major role as a main biological marker of host resistance during the cytokine response (49). The lower expression level of *CRP* in the liver of all the treatment groups suggests reduced systemic infection and liver inflammation. The reduced expression levels of *CRP* in the ASC-treated group compared to the infected-control and chlorine-treated groups suggest reduced invasion potential of C. jejuni following exposure to ASC. The reduced expression of *MBL1* either in the liver or spleen could be due to the reduced clinical infection in the present study. TLR pathways activate the *NF-κB1* inflammatory cytokine pathway in C. jejuni infection (50). Therefore, the upregulation of *NF-κB1*in infected control and chlorine-treated groups could be due to the increased inflammation followed by higher tissue colonization. Higher expression of *NF-κB1* has been demonstrated in a previous study with severe intestinal inflammation in IL-10^−/−^ mice (51). A recent study showed that NF-κB is involved in the mechanism of clearance of C. jejuni (52). In general, reduced expression of host stress response genes by sublethally injured C. jejuni after exposure to either chlorine or ASC in the present study suggested a reduced pathogenicity in mice. We previously demonstrated in a transcriptomic study the expression of genes related to the physiological and structural changes of the membrane in exposure to sanitizers in C. jejuni (29). Furthermore, our previous *in vitro* study showed overexpression of virulence and stress response genes (*racR*, *flgA*, *flaA*, *cadF*, *sodB*, *rpoB*) of sublethally injured C. jejuni after exposure to chlorine and ASC during storage in chicken meat juice. However, in the present *in vivo* study, we were not able to analyze the expression of virulence genes (*flaA*, *cadF*, *peb1*, *racR*, and *cheA*) via qPCR, possibly due to the undetectable level of bacterial RNA in host tissue. This could be due to the lower bacterial load. We have previously determined the optimized required bacterial concentration of C. jejuni to obtain quality RNA for a gene expression study as 10^9^ CFU/mL (29). Clinical disease was not observed in C57BL/6J-ARC mice we used in the present study. This further confirmed the limitations of using mouse models in Campylobacter clinical infection studies.

### Conclusions.

Results obtained in this study demonstrated that sublethally injured Campylobacter jejuni following exposure to either chlorine or ASC and storage in chicken meat juice was not able to cause clinical disease in mice, but it was still able to colonize the gut. The bacterial cell damage and the structural changes in bacterial membrane proteins following exposure to ASC may significantly reduce the intestinal colonization, tissue translocation, and expression of immune and host stress response genes. The data showed that ASC had better bactericidal effects than chlorine. The findings contribute toward establishing an effective chemical decontamination protocol and selection of a sanitizer to mitigate Campylobacter contamination in chicken meat necessary for reduction of human campylobacteriosis.

## MATERIALS AND METHODS

### Campylobacter inoculum preparation.

Campylobacter jejuni isolates obtained from chicken carcasses as part of a previous study (53) were maintained at −80°C in brain heart infusion broth and 50% glycerol. Two C. jejuni isolates, low invasive (C10) and high invasive (C5), were characterized in a previous study (21). The C5 C. jejuni isolate exhibited >10^5^ CFU/mL cell invasion into cultured Caco2 cells, while C10 exhibited <10^2^ CFU/mL invasiveness. C. jejuni isolates were resuscitated on Columbia sheep blood agar (SBA; Thermo Fisher Scientific, Australia) and incubated at 42°C for 48 h in 10% CO_2_.

An 8 ppm chlorine solution (composed of 4% sodium hypochlorite [Sigma-Aldrich, USA]) and an acidified 31% sodium chlorite solution (900 ppm, pH 2.4 to 2.5 [Chem-Supply, Australia]) were prepared in sterile water as previously described (54). Chlorine and ASC concentrations were chosen according to the Australian standard protocol for chemical decontamination in processing plants (53, 55). For each Campylobacter strain, a 10^10^-CFU/mL final bacterial concentration was prepared and exposed either to ASC for 1 min or chlorine for 2 min. Bacterial suspensions in 0.9% saline were not exposed to sanitizers and served as the infection controls. To prepare the inoculum, either chemically treated C. jejuni cultures or untreated controls were added to 3 mL of sterile chicken meat juice (CMJ) and stored at 5°C for 5 days. Sterile CMJ was prepared as previously described (21). The inoculum was confirmed on day 0 and day 5 by preparing 10-fold serial dilutions of CMJ. A 100-μL aliquot of each dilution was spread plated onto mCCDA agar plates (Thermo Fisher Scientific, Australia) and incubated at 42°C for 48 h in 10% CO_2_.

### Animals and treatment groups.

C57BL/6J-ARC male mice (4 weeks of age; *n *= 140) were obtained from the Animal Resources Centre in Perth, Western Australia. All mice were housed in groups of seven (*n *= 7) in polycarbonate microisolator cages with autoclaved bedding material. Sterilized water and food were provided *ad libitum*. The animal experiment was performed with the approval of the University of Adelaide Animal Ethics Committee (approval number S-2021-026) under the Australian Code for the Care and Use of Animals for Scientific Purposes. Separate groups were maintained for all three Campylobacter strains, ATCC 33291, C5, and C10.

### Experimental infection.

Mice were orally administered 100 μL of CMJ previously inoculated with either ATCC 33291, C5, or C10. Uninfected mice received 100 μL of sterile CMJ. Although the bacterial count (in CFU per milliliter) was different in the chicken meat juice on the day of inoculation after being exposed to chlorine and ASC, to mimic the natural setup to determine the effect of sublethally injured C. jejuni stored in chicken meat juice, we inoculated the exact amount of bacteria remaining in the chicken meat juice. Table 1 summarizes the experimental design.

**TABLE 1:** Experimental design

Design element	Infected control			Chlorine treated			ASC treated			CMJ control
	ATCC	C5	C10	ATCC	C5	C10	ATCC	C5	C10	
Bacterial inoculum before exposure to sanitizers (CFU/mL)	1 × 10^10^	1 × 10^10^	1 × 10^10^	1 × 10^10^	1 × 10^10^	1 × 10^10^	1 × 10^10^	1 × 10^10^	1 × 10^10^	0
Bacterial count after exposure to sanitizer (CFU/mL)	1 × 10^10^	1 × 10^10^	1 × 10^10^	2 × 10^8^	2 × 10^9^	8 × 10^5^	NC^*a*^	NC	NC	0
Bacterial count on day of infection or inoculum dose (CFU/mL)	2 × 10^9^	4 × 10^9^	1 × 10^9^	7 × 10^6^	2 × 10^7^	5 × 10^4^	NC	NC	NC	0
No. of mice in group	14	14	14	14	14	14	14	14	14	14
No. of mice euthanized day 3 p.i.	7	7	7	7	7	7	7	7	7	7
No. of mice euthanized day 7 p.i.	7	7	7	7	7	7	7	7	7	7

a NC, nonculturable.

Mice were monitored postinfection for clinical signs of disease, such as reluctance to move, hunching behavior, weight loss, and ruffled coat. Body weight was measured before infection (day 0) and on days 2 and 6 p.i. Fecal pellets were collected as a pooled sample from individual cages of all the groups on the same day for fecal Campylobacter detection.

### Fecal hemoglobin assay.

Fecal hemoglobin was measured for all treatment and control groups. Samples were prepared for the hemoglobin assay by suspending 1 mg of feces in 1 mL of sterile water. The samples were vortexed and centrifuged for 2 min at 8,000 × *g*. The supernatant was used to detect the fecal hemoglobin using the mouse hemoglobin ELISA kit (Abcam, Australia). Standard curve generation and sample testing were performed as per the manufacturer’s protocol. Each sample was tested in duplicate.

### Quantitation of colonization and tissue translocation of Campylobacter.

The mice were humanely euthanized on day 3 or day 7 p.i., and spleen, liver, ileal, colon, and, cecal tissue samples were collected and processed for total Campylobacter load. The tissue samples were placed into 0.5 mL of 0.9% saline in sterile lysis matrix tubes containing ceramic spheres (1.4 mm; MP Biomedicals, USA). Additional spleen, liver, and ileal samples were collected in RNAlater solution (Invitrogen, Australia) and stored in a −80°C freezer for RNA extraction.

The tissue samples were homogenized using a FasTPrep-24 5G sample preparation system (MP Biomedicals, USA) at 6.0 m/s speed for 40 s. Serial 10-fold dilutions of the homogenates were prepared, and 100 μL of each dilution was spread plated onto mCCDA agar and incubated at 42°C for 48 h in 10% CO_2_. Subsequently, 100 mg of fecal pellets was suspended in nutrient broth no. 2 (Thermo Scientific, Australia) and incubated at 42°C for 48 h in 10% CO_2_, followed by spread plating onto mCCDA. All culture-negative tissue samples and fecal pellets were resuscitated by enriching in nutrient broth no. 2, subsequent incubation at 42°C in 10% CO_2_ for 24 h, and plating onto mCCDA.

### Fecal and tissue DNA extraction and PCR for Campylobacter detection.

DNA was extracted from approximately 100 mg of feces using the QIAamp Fast DNA stool minikit (Qiagen, Australia) according to the manufacturer’s recommended protocol with some modifications, as previously described (56). DNA was extracted from 200 μL of tissue homogenate using a DNeasy blood and tissue kit (Qiagen, Australia) according to the manufacturer’s protocol. The DNA from all samples was tested for quality and quantity using a Nanodrop-1000 spectrophotometer (ThermoFisher, Scientific, Australia). The feces and tissue DNA samples were stored at −20°C until needed.

C. jejuni species-specific PCR (97 bp) was performed to detect the 16S rRNA gene (forward: 5′-TGCTAGAAGTGGATTAGTGG-3′; reverse: 5′-GTATTAGCAGTCGTTTCCAA-3′). The 20-μL final reaction volume contained 2 μL (100 ng) DNA, 4 μL of 1× My Red *Taq* reaction buffer, 0.2 units of My Red *Taq* polymerase (Bioline, Australia), 500 nM each of the forward and reverse primers, and water. The endpoint PCR was performed using the Bio-Rad T100 thermocycler cycling conditions previously described (28). The PCR amplicons were visualized by 2% agarose gel electrophoresis.

Fecal pellets were collected as pooled samples from each cage; therefore, the results are presented in positive proportion to the number of cages for each treatment. PCR-positive tissue samples are presented as a proportion of the number of all collected samples of the relevant tissue.

### RNA extraction from tissues.

To study the host immune response to sublethally injured C. jejuni, RNA was extracted from spleen, liver, and ileum of four mice in each group on day 3 and day 7 p.i. Total tissue RNA was extracted using TRIzol (Invitrogen, USA). Approximately 30 to 40 mg of tissue previously stored in RNALater was homogenized using an IKA T10 basic homogenizer (Wilmington, NC, USA) in 1 mL TRIzol reagent and 10 μL of β-mercaptoethanol (Merck, Australia). During homogenization, samples were maintained on ice and further incubated for an additional 7 min on ice. Subsequently, 0.2 mL of bromochloropropane (Merck, Australia) was added to each sample. The samples were incubated on ice for 5 min and centrifuged at 12,000 × *g* for 15 min at 4°C. The aqueous phase of the supernatant was transferred into 0.5 mL chilled (−20°C) isopropanol (Merck, Australia) and incubated for 1 h in a −20°C freezer. The samples were then centrifuged at 12,000 × *g* for 10 min at 4°C. The extracted RNA was washed in 1 mL of 75% ethanol, centrifuged, subsequently air dried for 5 to 10 min on ice, and resuspended in 50 μL water. The quality and quantity of RNA were measured with a Nanodrop instrument (ThermoFisher, Scientific, Australia) and stored at −80°C.

### Primer design and validation.

Genes for the mouse innate immune response (*IL-6*, *IL-10*, *IL-23a*, *TLR2*, and *TLR4*) and host stress response (*CRP*, *MBL1*, and *NF-κB1*) were selected for qPCR. In addition, C. jejuni virulence genes (*flaA*, *cadF*, *peb1*, *racR*, and *cheA*) were evaluated in a qPCR as previously described (21). Primer sequences of candidate target and reference genes were designed using the National Center for Biotechnology Information (NCBI) software (Table 2) and synthesized by Sigma-Aldrich, Australia. The target specificity was optimized by melt curve analysis with qPCR, and the amplicons were visualized using a 2% agarose gel. To determine the amplification efficiency (as a percentage) of individual primers, qPCR was performed on 5-fold serial dilutions of mouse tissue cDNA.

**TABLE 2:** Primer sequences used in qPCR

Gene	Primer sequence (5′–3′)	Fragment size (bp)	NCBI accession no.	Annealing temp (°C)	PCR efficiency (%)^*a*^	Correlation coefficient (*R*^2^)	Slope
*HPRT*	F: AGTCCCAGCGTCGTGATTAG; R: TGATGGCCTCCCATCTCCTT	170	NM_013556.2	55	105.5	0.995	−3.198
*β*-*Actin*	F: ACTGTCGAGTCGCGTCCA; R: ATCCATGGCGAACTGGTGG	86	NM_007393.5	55	105.8	1.00	−3.138
*IL-6*	F: TACCACTTCACAAGTCGGAGGC; R: CTGCAAGTGCATCATCGTTGTTC	116	X54542.1	55	110.2	0.997	−2.98
*IL-10*	F: TTGCCAAGCCTTATCGGAAA; R: CACCTTGGTCTTGGAGCTTATT	224	NM_010548.2	55	107.2	0.998	−3.16
*IL-23a*	F: CACCAGCGGGACATATGAATCT; R: AGACCTTGGCGGATCCTTTG	148	NM_031252.2	57	110.1	0.998	−2.99
*TLR2*	F: GAAACCTCAGACAAAGCGTCAAA; R: ACAGCGTTTGCTGAAGAGGA	98	NM_011905.3	55	102.1	0.999	−3.187
*TLR4*	F: AATCCCTGCATAGAGGTAGTTCC; R: TCAAGGGGTTGAAGCTCAGAT	121	NM_021297.3	55	97.6	0.999	−3.38
*CRP*	F: TCGGACTTTTGGTCATGAAGACAT; R: AGAGAAGACACTGAAGCTGCG	172	NM_007768.4	55	103.7	0.999	−3.236
*MBL1*	F: CAGGCTAATGGCCCTGTCAT; R: GAAGCATGGTCCTTACTAGGGT	112	NM_010775.2	55	102.3	0.99	−3.268
*NF-κB1*	F: GTCAAAATTTGCAACTATGTGGGG; R: AGGTTTGCAAAGCCAACCAC	167	AY521463.1	55	103.6	0.999	−3.184
*peb1*	F: ACAAGAGGCCCTTTGCTTGA; R: TAGTTGCAGCTTGAGCCACT	202	NC_002163.1	55	102.3	0.997	−3.268
*cheA*	F: GCGGCACCTAAACCGACTAA; R: ACCTCCACGCGTATGGTTTG	116	NC_002163.1	55	103.1	0.998	−2.249

a The amplification efficiency of an individual genes was determined by qPCR using 5-fold dilutions of cDNA amplified using a QuantStudio 6 thermocycler real-time system (ABI, Australia).

### cDNA synthesis and reverse transcription-qPCR.

Approximately 1,000 ng of individual RNA samples (*n *= 240) were subjected to cDNA synthesis using the manufacturer’s protocol for the QuantiTect reverse transcription kit (Qiagen, Australia). The master mix was prepared using a SensiFast Sybr Hi-Rox kit (Bioline, Australia), and qPCR was performed in QuantStudio 6 (ABI, Australia) as previously described (54). Annealing temperatures used in the cycling conditions are listed in Table 2. The relative expression data were analyzed by the 2^−ΔΔCq^ method using *HPRT* and *β*-*actin* as reference genes. The untreated control group was used as a reference in assessing the expression of genes of C. jejuni affected by chlorine and ASC in mice. All samples were run in duplicates with four biological replicates. The data are expressed in a fold change format.

### Statistical analysis.

All the data were statistically analyzed by either a one-way or two-way ANOVA with Tukey’s multiple-comparison test and Student’s *t* test, within GraphPad Prism version 9 (GraphPad Software, Inc., USA). The level of significance between the treatment groups was determined at a *P* value of ≤0.05.
